# Long term follow-up in two siblings with Sengers syndrome: Case report

**DOI:** 10.1186/s13052-022-01370-y

**Published:** 2022-10-17

**Authors:** Chiara Panicucci, Maria Cristina Schiaffino, Claudia Nesti, Maria Derchi, Gianluca Trocchio, Mariasavina Severino, Nicola Stagnaro, Enrico Priolo, Federico Zara, Filippo M. Santorelli, Claudio Bruno

**Affiliations:** 1 Center of Translational and Experimental Myology, IRCCS Istituto Giannina Gaslini, Via G. Gaslini, 5, I-16147 Genova, Italy; 2 Pediatric Clinic Unit, IRCCS Istituto Giannina Gaslini, Genova, Italy; 3 Molecular Medicine, IRCCS Stella Maris Foundation, Pisa, Italy; 4 Cardiology Unit, IRCCS Istituto Giannina Gaslini, Genova, Italy; 5 Neuroradiology Unit, IRCCS Istituto Giannina Gaslini, Genova, Italy; 6 Radiology Unit, IRCCS Istituto Giannina Gaslini, Genova, Italy; 7 Ophthalmology Unit, IRCCS Istituto Giannina Gaslini, Genova, Italy; 8 Medical Genetics Unit, IRCCS Istituto Giannina Gaslini, Genova, Italy; 9 Department of Neurology, Rehabilitation, Ophthalmology, Genetics, Maternal and Child Health, University of Genova, Genova, Italy

**Keywords:** Sengers syndrome, *AGK* gene, Mild phenotype, Long term follow-up, Case report

## Abstract

**Background:**

Sengers syndrome is characterized by congenital cataract, hypertrophic cardiomyopathy, mitochondrial myopathy, and lactic acidosis associated with mutations in *AGK* gene. Clinical course ranges from a severe fatal neonatal form, to a more benign form allowing survival into adulthood, to an isolated form of congenital cataract. Thus far few reported cases have survived the second decade at their latest examination, and no natural history data are available for the disease.

**Case presentation:**

Here we provide a 20-year follow-up in two siblings with a benign form of Sengers syndrome, expanding the phenotypical spectrum of the disease by reporting a condition of ovarian agenesis.

**Conclusion:**

To our knowledge, this report provides the first longitudinal data of Sengers syndrome patients.

**Supplementary information:**

The online version contains supplementary material available at 10.1186/s13052-022-01370-y.

## Background

Sengers syndrome (SS, OMIM #212,350), is a rare autosomal recessive disorder described in 1975 with a prevalence < 1/1,000,000 [1]. It is clinically characterized by congenital cataract, hypertrophic cardiomyopathy, mitochondrial myopathy, and lactic acidosis, and genetically by mutations in the acylglycerol kinase (*AGK*) gene [2]. *AGK* codes for a mitochondrial protein, also known as multi-substrate lipid kinase (MULK), located in the inner mitochondrial membrane, and involved in the conversion of monoacylglycerol (MAG) and diacylglycerol (DAG) to lysophosphatidic acid (LPA) and phosphatidic acid (PA), respectively [3]. Independently of its lipid kinase activity, AGK is also required for the import and assembly of mitochondrial carrier proteins in the inner membrane [4], thus indicating that pathogenic mechanisms for SS rely both on defective lipid metabolism [2] and protein biogenesis in mitochondria [4].

Clinical course ranges from a severe fatal form, leading to death within the third year of age in around 86% of cases [8], to a more benign form with survival into adulthood [7], to an isolated form of congenital cataract [9]. To date, only 2/44 genetically reported patients [2–23] have survived the second decade at their latest examination [10, 11], and no natural history data are available for the disease.

Here we report a 20-year follow up in two Italian siblings with a benign form of Sengers syndrome associated with a homozygous splicing mutation in *AGK*. To our knowledge, this report provides the first longitudinal data of SS patients.

### Case Presentation

**Case 1** Case 1 is a Caucasian female, coming from Sardinia, Italy. She is the second child of consanguineous parents (third cousins), born at term after an uneventful pregnancy. Her weight at birth was 2450 g, and she required an invasive respiratory support for few days after birth because of a transitory respiratory distress. Global neurodevelopmental delay was noted, encompassing speech and motor delay with autonomous ambulation reached at 24 months. Congenital bilateral cataract was diagnosed and surgically corrected at 5 months of age.

At first examination, at 9 years, a global growth delay was noted. Height and weight were below the 3° percentile of CDC growth charts. She presented behavioral disturbances with aggressiveness. Neurological exam showed cognitive delay, bilateral nystagmus and convergent strabismus. Muscle tone and strength were normal. Clumsiness and hyperactive bilateral deep tendon reflexes were present.

Laboratory findings showed a metabolic acidosis (venous pH 7.29, reference ranges: 7.35–7.45) with mild increase of serum lactate (3.14 mmol/L, reference ranges: 0.5-2.0 mmol/L). An extensive metabolic work-up showed high urine levels of lactic and pyruvic acid, with normal blood carnitine.

At electrocardiogram (ECG), left ventricular hypertrophy signs were detected, including markedly high voltage R waves, ST interval depression with negative T waves in lead I, inferior and left lateral leads, and non-ischemic ST elevation, as well as positive T wave in aVR. Echocardiography showed normal cardiac dimensions with a maximum septal thickness to the upper limit of the normal range (6 mm), associated with a conserved Left Ventricular Ejection Fraction (LVEF) measured, with no outflow obstruction.

**Fig. 1:**
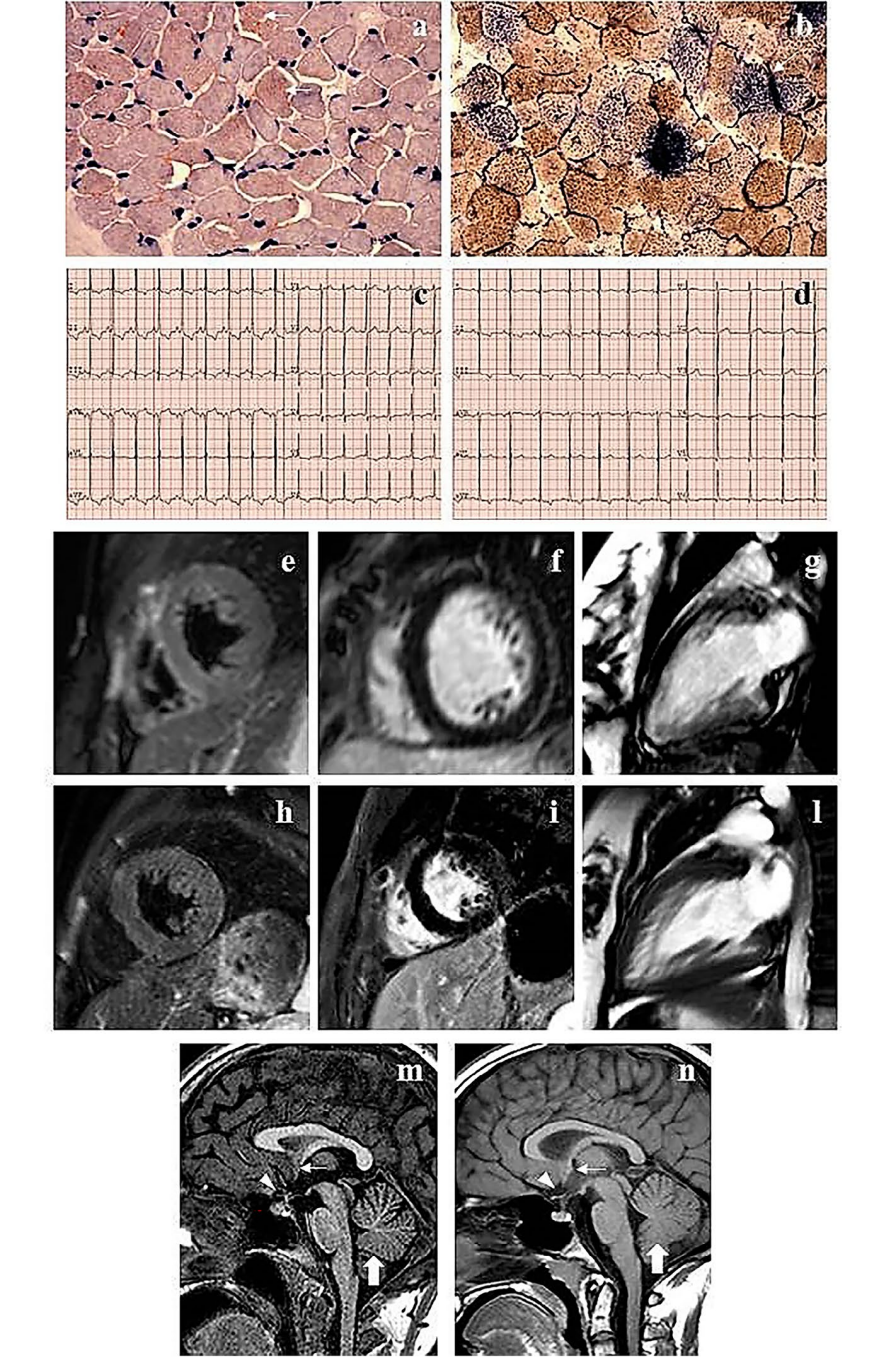
**Histochemical, cardiological and neuroradiological findings** Muscle biopsy studies performed in case 1. With the Oil red O (ORO), many fibers displayed lipid droplets (arrows) (**a**). The combine staining for oxidative enzymes showed several fibers COX negative and SDH strongly positive (arrows) (**b)**. ECG performed at last examination in case 1 (25 years) (**c**) and in case 2 (17 years) (**d**): left ventricular hypertrophy signs were detected, including markedly high voltage R waves, non-ischemic ST segment alterations with negative T waves in lead I, inferior and left lateral leads, as well as positive or isodiphasic T wave in aVR. Cardiac MRI (1,5 Tesla) performed in case 1 at the age of 19 years showed normal myocardial appearance and signal intensity: STIR short axis (**e**), PSIR (Late Gadolinium Enhancement) (**f**) and Balanced Turbo Field-Echo (BTFE) cine 2 chamber left **(g).** Cardiac MRI performed in case 2 at the age of 16 years showed normal myocardial signal intensity: STIR short axis (**h**) and PSIR (Late Gadolinium Enhancement) (**i**). A hypertrabeculation of mid and apical segments partially ascribed to multihead papillary muscles attachment to the endocardial surface of LV free wall was noted: BTFE cine 2 chamber LV (**l**). Brain MRI, sagittal T1-weighted images of case 1, at the age of 22 (**m**), and case 2, at age of 12 (**n**), revealed marked thinning of the optic chiasm (arrowheads), hypoplasia of the anterior commissure (arrows), and mild inferior vermis hypoplasia (thick arrows)

Since clinical and biochemical findings suggested a mitochondrial disorder, a muscle biopsy was performed at 9 years of age. A mild fiber diameter variability was noted, and several fibers displayed small intracytoplasmatic vacuoles at the Trichromic Gomori staining (TG), with sporadic fibers presenting larger vacuoles. Several fibers displayed lipid droplets with the Oil red O (ORO) staining (Fig. 1a). Staining for oxidative enzymes showed cytochrome c oxidase (COX) negative fibers strongly positive to succinate dehydrogenase (SDH) (Fig. 1b). Respiratory chain enzyme activities in total muscle homogenate showed a decreased nicotinamide-adenine dinucleotide dehydrogenase (NADH) and COX complex activities (Complex I and IV).

The results of muscle biopsy, together with the presence of cataract, lactic acidosis and left ventricular hypertrophy, prompted us to analyze the *AGK* gene. Sanger sequencing revealed a homozygous variant affecting the donor splice-site of intron 4 (c.221 + 1G > A), previously reported [2].

From the age of 9, she was started with daily oral sodium bicarbonate, and at 12 years, she presented with right retinal detachment which occurred few years later in the contralateral eye. Drospirenone/ethinylestradiol therapy was started at 14 years to threat a pubertal delay condition due to hypergonadotropic hypogonadism which was diagnosed by a delayed bone age and a pathological gonadotropin-releasing hormone (GnRH) test. A pelvic ultrasound failed to detect ovaries, indicating a diagnosis of ovarian agenesis.

ECG abnormalities persisted along the following years (Fig. 1c), while no arrhythmias were detected on Holter ECG. Annual echocardiographic follow-up showed mild evolutive not obstructive hypertrophic cardiomyopathy with a globally conserved sisto-diastolic function, and a global longitudinal strain peak (GLS) at lower limits, particularly in the septal regions. Cardiac MRI performed at 19 years showed normal left and right ventricular volumes with a preserved systolic function (LVEF 56%), normal LV mass, and no outflow obstruction. Additionally, a hypertrabeculation of inferior and lateral walls was noticed. No abnormalities were noted on T1, T2, Short-TI Inversion Recovery (STIR) sequences, as well as no myocardial Late Gadolinium Enhancement (LGE) was appreciated (Fig. 1e-g).

Brain MRI performed at 23 years of age revealed marked thinning of the optic nerves and chiasma, hypoplasia of the anterior commissure, and mild hypoplasia of the inferior cerebellar vermis (Fig. 1 m). No lactic acid peaks were noted at MR spectroscopy (MRS).

At last examination, at age 29, the patient was neurologically stable, with a severe cognitive delay, not quantifiable with the available cognitive assessments, and requiring constant support by caregivers. Blood examinations showed high serum lactate levels 3.96 mmol/L with venous pH within the normal ranges and no other biochemical abnormalities. Platelet count has always being within the normal ranges during the follow-up period and no hemorrhagic or thrombotic complications have been observed.

**Case 2** The youngest sister of case 1, was born at 35 weeks of gestational age after an uneventful pregnancy; her weight at birth was 2008 g. Nystagmus, convergent strabismus and congenital bilateral cataract were diagnosed at birth, and the cataract was surgically corrected at the age of 6 months. Failure to thrive was observed since her first months of life, together with a global neurodevelopmental delay.

Considered the family history and her clinical condition, Sengers Syndrome was suspected, and gene testing showed the same homozygous c.221 + 1G > A variant in *AGK* identified in the sister.

At 1 year of age physical examination showed height, weight and cranial circumference measurements below the 10° percentile of CDC growth charts, trigonocephaly with ocular hypotelorism and 2/6 systolic murmur. Neurological examination revealed generalized hypotonia, normal deep tendon reflexes, mild delay of motor milestones, bilateral nystagmus, and convergent strabismus. Brain MRI did not show abnormalities, with normal white matter myelination for the age. Serum lactate level was 3.89 mmol/L (reference ranges: 0.5-2.0 mmol/L); urine analysis showed high urine levels of lactic acid.

ECG showed signs of ventricular hypertrophy, i.e., markedly high voltage R waves, negative T waves in inferior and left lateral leads and positive T wave in aVR. Echocardiography revealed left ventricular posterior wall and papillary muscles hypertrophy with normal interventricular septum thickness (4 mm), trabeculations on the posterolateral wall of the left ventricle was also noted, LVEF was 60%, and no outflow obstruction was observed.

From the age of 4 years, the patient was supplemented with daily oral sodium bicarbonate.

Estradiol therapy was started at 14 years to threat pubertal delay due to hypergonadotropic hypogonadism, and a pelvic MRI documented ovarian agenesis.

ECG abnormalities persisted along the follow up, showing signs of left ventricular hypertrophy (Fig. 1d), with no arrhythmias at Holter ECG. Follow-up cardiac ultrasounds showed a slowly evolutive and not obstructive hypertrophic cardiomyopahty. Cardiac MRI performed at 13 years showed normal left and right ventricular volumes associated with a preserved systolic function; LV mass was normal, and no outflow obstruction was documented. No abnormalities were noted on T1, T2, STIR sequences, as well as no myocardial late enhancement was appreciated. At age 16 a follow-up cardiac MRI was performed. LEFV was 79%. Mild hypertrophy of the interventricular septum was measured (z-score + 2.7) and a fine left ventricle inferior-lateral hypertrabeculation was noticed (Fig. 1 h-l).

Follow-up brain MRI, performed at 13 years of age, denoted marked thinning of the optic nerves and chiasma, hypoplasia of the anterior commissure, and mild hypoplasia of the inferior cerebellar vermis (Fig. 1n), without lactic acid peaks at MRS.

Latest neurological examination at age 20 was remarkable for mild axial hypotonia, mild muscle hypotrophy, slightly reduced strength in the legs and hyperactive deep tendon reflexes. Strabismus, nystagmus and clumsiness were still present, combined with a cognitive delay. The Wechsler Adult Intelligence Scale-Revised (WAIS-R) showed a total intelligence quotient (TIQ) of 61 (average 85–115, low average 70–85, below average 55–70), a verbal intelligence quotient (VIP) of 84 (average 85–115, low average 70–85, below average 55–70), and a performance intelligence quotient (PIQ) below 45 (average 85–115, low average 70–85, below average 55–70), that was highly affected by her visual disability. Overall she was neurologically stable and independent in her daily activities, despite her cognitive delay. Blood examination showed high lactic acid values (43 mg/dl) with venous pH within the normal ranges and no other biochemical abnormalities. Normal platelet count was observed throughout the follow-up and no bleeding or thrombotic complications have been reported.

## Discussion and conclusions

We present a long term follow up in two siblings affected by a slowly progressive benign form of SS. The association of congenital cataract, lactic acidosis, mitochondrial myopathy, and a mild hypertrophic cardiomyopathy suggested the diagnosis which was confirmed by the identification of pathogenic mutations in *AGK* gene, which codes for a protein involved in lipid metabolism and protein biogenesis in mitochondria. Consistently, muscle biopsy highlighted mitochondrial abnormalities, associated with impaired Complex I and IV activities in muscle homogenate, in accordance with previous reports [6, 7, 12].

We have been monitoring the cardiological evolution of both sisters for 20 years, with ECG, echocardiography, Holter monitoring and cardiac MRI, providing evidence of a mild stable form of hypertrophic cardiomyopathy without outflow obstruction and absence of arrhythmic events, not requiring pharmacological intervention, thus far. We underline the significant discrepancy between pronounced abnormality and severity of electrical features on ECG and the effective grade of ventricular hypertrophy documented by both echo and cardiac MRI. The severity of cardiomyopathy is the main clinical predictor of disease progression in SS. Indeed, the detection of outflow obstruction at diagnosis is usually associated with the severe form of the syndrome, and its development during the clinical course worsens the outcome in the milder form [11]. The absence of outflow obstruction, and the overall conserved cardiac function in our patients, suggested a mild form of SS.

In both cases, brain MRI failed to detect major cerebral, cerebellar, and brainstem abnormalities, revealing only mild hypoplasia of the inferior cerebellar vermis and anterior commissure in both siblings. Moreover, the white matter myelination was adequate for the age and MRS showed normal metabolic profiles. Of note, thinning of the optic nerves and chiasm was a consequence of the longstanding ocular problems. Neuroradiological examinations have been showed in around 30% of reported cases [1, 6, 16, 17, 19, 24], with the most common findings represented by hypoplasia of both the brainstem and inferior cerebellar vermis, impaired myelination of the cerebral hemispheres and brainstem, and cortical infarction. It has been suggested that the presence of radiological alterations might discriminate the severe form from the mild one [24]. Recent studies and our findings support this hypothesis, showing grossly normal brain MRI in mild form of SS [2, 17, 19, present study].

Our patients also presented a motor development delay in their infancy and a moderate to severe cognitive delay. While the first has been reported in 22 out of 23 patients [20], conversely, cognitive delay is rarely reported [14, 17] and its burden on the daily life of those who survive until the adult age is not known and should be studied in further studies. The milder cognitive impairment in the younger sister might be explained with an earlier diagnosis and initiation of a multidisciplinary rehabilitation program compared to her sister.

Both our patients presented with hypergonadotrophic hypogonadism related to ovarian agenesis, a disorder which has not been previously reported in SS, though premature ovarian failure was reported in a single case [15]. Since peripheral and central hypogonadisms are common in several mitochondrial diseases [25], and AGK is expressed in the ovarian and testis tissue [The Human Protein Atlas; available from: https://www.proteinatlas.org/ENSG00000006530-AGK/tissue], the prevalence of hypogonadism might be underestimate in SS, suggesting that a work-up for an early recognition and treatment of this condition should be performed in those patients who survive the first decade.

A clear-cut genotype-phenotype correlation for SS has not been clearly defined yet, probably due to the few cases so far reported. However, a more severe phenotypes have been described in homozygous or compound heterozygous carriers of nonsense variants [8], whether all patients who survived the first decade, retained at least one splice site variant [7, 9]. Our patients harbored a homozygous splicing variant on the AGK gene, already described in compound heterozygosity with the nonsense c.1213 C > T/ p.Gln405* (see case 62,218 in [2]). The previously reported child displayed failure to thrive, progressive muscle weakness requiring wheelchair for distances longer than 100 m from 12 years on, and a cardiac condition treated with propranolol and idebenone. Our cases confirm that splicing variants are associated to milder forms, in particular when in homozygous state. However, genotype-phenotype relationships in SS remain loose. Indeed, two homozygous carriers of the c.424-1G > A splicing variant died at 10 days and 4 months, because of severe heart dysfunction [7], and an infant harboring the homozygous c.518 + 1G > A [22] died soon after birth for congestive heart failure.

In conclusion, we defined for the first-time the natural history of Sanger Syndrome in two siblings with a mild form of SS, across a 20-years follow-up. During this period, the patients showed an extremely slowly evolutive hypertrophic cardiomyopathy, a mild and stable myopathy associated to cognitive delay, and a condition of ovarian agenesis, not previously reported. We emphasize the importance of natural history studies on SS necessary to future clinical trial readiness.

## Electronic supplementary material

Below is the link to the electronic supplementary material.

Supplementary Material 1

Supplementary Material 2

## Data Availability

**T**he datasets used and/or analysed during the current study are available from the corresponding author on reasonable request.

## List of abbreviations

AGK: acylglycerol kinase.
BTFE: Balanced Turbo Field-Echo.
CDC: Clinical Growth Charts.
COX: cytochrome c oxidase.
DAG: diacylglycerol.
ECG: electrocardiogram.
GnRH: gonadotropin-releasing hormone.
LGE: Late Gadolinium Enhancement.
LPA: lysophosphatidic acid.
LVEF: Left Ventricular Ejection Fraction.
MAG: monoacylglycerol.
MRI: Magnetic Resonance Imaging.
MULK: multi-substrate lipid kinase.
NADH: nicotinamide-adenine dinucleotide dehydrogenase.
ORO: Oil red O.
PA: phosphatidic acid.
PIQ: performance intelligence quotient.
SDH: succinate dehydrogenase.
SS: Sengers Syndrome.
STIR: Short-TI Inversion Recovery.
TG: Trichromic Gomori.
TIQ: total intelligence –quotient.
VIP: verbal intelligence quotient.
WAIS-R: Wechsler Adult Intelligence Scale – Revised.
