# Supplementary material for: Long term follow-up in two siblings with Sengers syndrome: Case report
Source: Ital J Pediatr. 2022 Oct 17;48:180. doi: 10.1186/s13052-022-01370-y (PMC9575244; doi:10.1186/s13052-022-01370-y)
Supplement: Supplementary file 1 — Supplementary Material 1 [file 13052_2022_1370_MOESM1_ESM.pdf]

CARE case report guidelines

CARE Checklist of information to include when writing a case report

(cc) BY-NC-ND

✓

Topic	Item	Checklist item description	Reported on Line
Title	1	The diagnosis or intervention of primary focus followed by the words "case report"	2
Key Words	2	2 to 5 key words that identify diagnoses or interventions in this case report, including "case report"	33
Abstract (no references)	3a	Introduction: What is unique about this case and what does it add to the scientific literature?	30 - 31
	3b	Main symptoms and/or important clinical findings	29
	3c	The main diagnoses, therapeutic interventions, and outcomes	28
	3d	Conclusion—What is the main "take-away" lesson(s) from this case?	30 - 31
Introduction	4	One or two paragraphs summarizing why this case is unique (**may include references**)	48 - 50
Patient Information	5a	De-identified patient specific information.	53 + 101
	5b	Primary concerns and symptoms of the patient.	55 + 101 - 104
	5c	Medical, family, and psycho-social history including relevant genetic information	53 + 101
	5d	Relevant past interventions with outcomes	53 - 57 + 102 - 103
Clinical Findings	6	Describe significant physical examination (PE) and important clinical findings.	58 + 107
Timeline	7	Historical and current information from this episode of care organized as a timeline	
Diagnostic Assessment	8a	Diagnostic testing (such as PE, laboratory testing, imaging, surveys).	62 - 95 + 110 - 131
	8b	Diagnostic challenges (such as access to testing, financial, or cultural)	
	8c	Diagnosis (including other diagnoses considered)	77 - 78 + 105 - 108
	8d	Prognosis (such as staging in oncology) where applicable	
Therapeutic Intervention	9a	Types of therapeutic intervention (such as pharmacologic, surgical, preventive, self-care)	100 + 118
	9b	Administration of therapeutic intervention (such as dosage, strength, duration)	100 + 118
	9c	Changes in therapeutic intervention (with rationale)	
Follow-up and Outcomes	10a	Clinician and patient-assessed outcomes (if available)	
	10b	Important follow-up diagnostic and other test results	86 - 92 - 119 - 121 + 123-[illegible]
	10c	Intervention adherence and tolerability (How was this assessed?)	
	10d	Adverse and unanticipated events	
Discussion	11a	A scientific discussion of the strengths AND limitations associated with this case report	
	11b	Discussion of the relevant medical literature **with references**.	142 - 182
	11c	The scientific rationale for any conclusions (including assessment of possible causes)	
	11d	The primary "take-away" lessons of this case report (without references) in a one paragraph conclusion	184 - 188
Patient Perspective	12	The patient should share their perspective in one to two paragraphs on the treatment(s) they received	
Informed Consent	13	Did the patient give informed consent? Please provide if requested	Yes ☒ No ☐
