# Supplementary material for: Long term follow-up in two siblings with Sengers syndrome: Case report
Source: Ital J Pediatr. 2022 Oct 17;48:180. doi: 10.1186/s13052-022-01370-y (PMC9575244; doi:10.1186/s13052-022-01370-y)
Supplement: Supplementary file 2 — Supplementary Material 2 [file 13052_2022_1370_MOESM2_ESM.pdf]

UOSD Centro di Miologia Traslazionale e Sperimentale

Genova, July 29th 2022

Journal of Pediatrics
Editorial Office

RE: Manuscript # ITJP-D-22-00102 "Long term follow-up in two siblings with Sengers Syndrome: case report"

Dear Editor,

we are grateful for your suggestions and Referees' comments. We addressed all the points raised by Referees and we detailed our point-by point replies, also visible in the manuscript with the "track-changes" word mode.

Reviewer #1
Is there an idea of the disease frequency?
Sengers syndrome is a rare disorder with a prevalence <1/1000000.
We therefore added the following sentence at **Page 3 Line 35**: "Sengers syndrome (SS, OMIM #212350), is a rare autosomal recessive disorder described in 1975 with a prevalence <1/1000000".
Which is the ethnic origin of the family?
The family is originally from Sardinia, Italy.
We therefore added the following sentence at **Page 3 Line 53**: "Case 1 is a Caucasian female from Sardinia, Italy. She is the second child of consanguineous parents (third cousins), born at term after an uneventful pregnancy".

Reviewer #2
The description of platelet count and function in the two patients would be interesting.
In both patients platelet count was always within the normal range. We therefore added the following sentences at **Page 5 Line 105**: "Platelet count has always being within the normal ranges during the follow-up period and no haemorrhagic or thrombotic complications have been observed", and at **Page 6 Line 148**: Normal platelet count was observed during throughout the follow-up and no bleeding or thrombotic complications have been reported.
The order of figure mentioned in the text should be reviewed.
Case 2 brain MRI (Fig. 1 n) has been moved after cardiac MRI, resembling Case 1 assessment order at **Page 6 Lines 137-139** "Follow-up brain MRI, performed at 13 years of age, denoted marked thinning of the optic nerves and chiasma, hypoplasia of the anterior commissure, and mild hypoplasia of the inferior cerebellar vermis (Figure 1 n), without lactic acid peaks at MRS".

To the Editor
Missing the affiliation number 8 in superscript, please verify.
Number 8 superscript has been added on **Page 1 Line 12**.

Under the "Ethics approval and consent to participate", please provide the missing statement below.

- *A statement to confirm that all experimental protocols were approved by a name of the ethics committee and the committee's reference number if appropriate. If a study has been granted an exemption from requiring ethics approval, this should also be detailed in the manuscript (including the name of the ethics committee that granted the exemption).*

The study does not include any experimental protocol. Therefore this statement does not apply to our case report.

- *A statement to confirm that all methods were performed in accordance with the ethical standards as laid down in the Declaration of Helsinki and its later amendments or comparable ethical standards.*

We provided a sentence at **Page 11 Line 251:** **"**All procedures were performed in accordance with the ethical standards as stated in the Declaration of Helsinki" (it has been removed from **Page 6 Line 150**).

We believe we adequately addressed all Referees' concerns on the previous version of the manuscript and we hope you will find the revised paper acceptable for publication in The Italian Journal of Pediatrics.
Thank you for your consideration, we are looking forward to hearing from you.
Sincerely yours,

Claudio Bruno

Claudio Bruno, MD, PhD
Center of Translational and Experimental Myology
IRCCS Istituto Giannina Gaslini
Via G. Gaslini, 5
I-16147 Genova Italy
Phone: +39-010-56362756
mail: claudio2246@gmail.com
